# Comparing subjective intoxication with risky single-occasion drinking in a European sample

**DOI:** 10.1371/journal.pone.0241433

**Published:** 2020-11-17

**Authors:** Carolin Kilian, Jakob Manthey, Jacek Moskalewicz, Emanuele Scafato, Lidia Segura García, Janusz Sieroslawski, Jürgen Rehm

**Affiliations:** 1 Institute of Clinical Psychology and Psychotherapy, Technische Universität Dresden, Dresden, Germany; 2 Centre for Interdisciplinary Addiction Research, University of Hamburg, Hamburg, Germany; 3 Department of Psychiatry, Medical Faculty, University of Leipzig, Leipzig, Germany; 4 Institute of Psychiatry and Neurology, Warsaw, Poland; 5 National Observatory on Alcohol, WHO Collaborating Centre Research & Health Promotion on Alcohol and Alcohol-Related Health Problems, Istituto Superiore di Sanità, Rome, Italy; 6 Program on Substance Abuse, Public Health Agency of Catalonia, Barcelona, Spain; 7 Institute for Mental Health Policy Research, Centre for Addiction and Mental Health, Toronto, Ontario, Canada; 8 WHO Collaboration Centre, Centre for Addiction and Mental Health, Toronto, Ontario, Canada; 9 Institute of Medical Science, University of Toronto, Toronto, Ontario, Canada; 10 Campbell Family Mental Health Research Institute, Centre for Addiction and Mental Health, Toronto, Ontario, Canada; 11 Department of Psychiatry, University of Toronto, Toronto, Ontario, Canada; 12 I. M. Sechenov First Moscow State Medical University (Sechenov University), Moscow, Russian Federation; University of Jyvaskyla, FINLAND

## Abstract

In most epidemiological literature, harmful drinking—a drinking pattern recognized as closely linked to alcohol-attributable diseases—is recorded using the measure risky single-occasion drinking (RSOD), which is based on drinking above a certain quantity. In contrast, subjective intoxication (SI) as an alternative measure can provide additional information, including the drinker’s subjective perceptions and cultural influences on alcohol consumption. However, there is a lack of research comparing both. The current article investigates this comparison, using data from the Standardized European Alcohol Survey from 2015. We analysed the data of 12,512 women and 12,516 men from 17 European countries and one region. We calculated survey-weighted prevalence of SI and RSOD and compared them using Spearman rank correlation and regression models. We examined the role of the required quantity of alcohol needed for the drinker to perceive impairments and analysed additional demographic and sociodemographic characteristics as well as drinking patterns. In the most locations, the prevalence of SI was lower or equal to the prevalence of RSOD. Both prevalence estimates were highly correlated. Almost 8% of the variance in the difference between the individual-level frequencies of the SI and RSOD measures was explained by the individual quantity of alcohol needed to perceive impairments. Sociodemographic characteristics and drinking patterns explained less than 20% in the adjusted perceived quantity of alcohol needed. In conclusion, our results indicated that subjective measures of intoxication are not a preferable indicator of harmful drinking to the more conventional measures of RSOD.

## Introduction

Alcohol consumption is a major risk factor for the burden of disease and mortality in European adults [1, 2]. Different patterns of drinking contribute to this burden, including risky single-occasion drinking (RSOD), usually defined as drinking more than 60 grams of pure ethanol on one single occasion [3]. This definition was derived from risk functions, i.e., it constitutes the threshold above which the risk for nearly all diseases causally linked to alcohol use is elevated. These drinking levels are further thought to roughly correspond to the phenomenon of intoxication. Per definition, RSOD elevates the risk for a multitude of diseases and injuries [1], however, there is far less evidence for the subjective state of intoxication, except for an elevated risk of injuries for oneself and others [4, 5]. Compared to RSOD, however, occasions where drinkers felt intoxicated, may be memorised more accurately, and thus may be used to improve alcohol assessment and therefore screening performance (for the relevance of assessing large amounts of alcohol intake in screening instruments, see [6]). For prevention purposes and alcohol policy formulation, the investigation of patterns, causes, characteristics and consequences of subjective intoxication (SI) and its correspondence to RSOD appear to be worthwhile.

For burden calculation, RSOD is preferred to a SI measure, as the former can be more objectively assessed (e.g., “How often have you had 6 drinks or more on one occasion?”) and most of the epidemiological literature uses such quantity measures [7]. Conversely, subjective measures of intoxication are based on perceptions of intoxication (e.g., “How often did you drink enough to feel unsteady on your feet?”). Although objective measures enable more comparability, subjective data can offer additional information about the drinker’s subjective perceptions, about cultural and environmental influences [8, 9], and about interindividual differences, which may be based on tolerance and alcohol metabolism [10]. Moreover, as intoxication is perceived, it has an impact on health-relevant behaviours, such as driving under the influence of alcohol [11]. To establish a link between the two measures, surveys sometimes ask about the level of alcohol necessary to feel intoxicated.

There are only a few studies that have investigated SI from an epidemiological perspective. Thus, it was shown that the threshold of alcohol intake to perceive impairments changed over a 21-year time period in an US sample, indicated by a decrease in the number of drinks needed to feel intoxicated [9]. Furthermore, women, older adults and individuals with higher educational achievement were more likely to report SI at lower thresholds of alcohol exposure [9, 12]. With regard to Europe, SI varied between countries and geographical regions in samples of adolescents [13] and adults [14]. Using data from the Standardised European Alcohol Survey (Joint Action on Reducing Alcohol-Related Harm RARHA SEAS), which surveyed more than a dozen European countries, the threshold of alcohol intake at which adults perceive impairments was less than one third for British drinkers compared to Croatian drinkers [14]. Additionally, the authors identified a north-south gradient, which indicated a higher prevalence of SI in the northern countries of Europe compared to lower rates in the south. However, there is a lack of research comparing SI measures with conventional methods such as RSOD assessment.

In the current research, which is also based on the RARHA SEAS [15], we investigated both measures of intoxication, SI and RSOD, in order to determine the more appropriate survey measure for harmful alcohol use. We first calculated and compared the prevalence of SI with the RSOD based prevalence estimates by location, and then examined the quantity of alcohol needed for the drinker to perceive impairments. We analysed different indicators, which were likely to explain variance within the deviation of the subjective threshold (i.e., the quantity of alcohol needed to perceive impairments) from the gender-specific RSOD threshold value (i.e., 40 grams of pure ethanol in women, 60 grams of pure ethanol in men). We also investigated demographic variables, body weight, individual-level socioeconomic status, frequency and quantity of alcohol consumption, occurrence of RSOD, high-risk drinking (i.e. drinking above a defined threshold on every drink day), and preferred beverage type.

## Methods

In the RARHA SEAS survey, the question of ethical approval was left to the discretion of individual countries, as the regulations differ across Europe. Verbal informed consent was required from each respondent after a standard introduction was read to them. The introduction informed them about the study aims, funding, its voluntary character, its anonymity and the option of refusing to respond to any of the questions. No minors below 18 years old were sampled to participate in the survey.

### Data sources

Data were obtained from the RARHA SEAS conducted in 2015 in which more than 30,000 adults (18–65 years) from 19 European countries and one region participated. In all surveys, randomised sampling procedures were applied to select general population samples, with the mode of survey administration varying across countries (for details on survey methodology, see [15]).

To estimate alcohol consumption levels, a beverage-specific quantity frequency approach was applied to measure the usual intake of three basic beverage types (beer, wine, and spirits) during the past 12 months. Respondents were asked questions regarding the frequency of RSOD, assuming thresholds of 40 grams of pure ethanol for women and 60 grams for men. Both measures were combined to estimate the overall annual consumption of pure ethanol, from which average daily alcohol intake was then determined based on the reported generic frequency of drinking over the past 12 months. In order to avoid overestimation of individual consumption, capping procedure was applied at 0.5 litres of pure ethanol consumed per drink day and 182 litres per annum. SI was measured by the question ‘How often in the past 12 months did you drink enough to feel unsteady on your feet or so your speech was slurred?’. Additionally, respondents were asked a question regarding the number of drinks needed to achieve intoxication. The reference period for all questions was the past 12 months.

The frequencies of SI and RSOD occasions were assessed for all locations. However, for both Norway (*n* = 1,493) and Romania (*n* = 1,500), more than 5% of respondents did not answer the SI item and therefore, individuals from these countries were excluded from the analyses. Individuals, who indicated that they consumed alcohol during the past 12 months and who reported any frequency of SI were included in the analyses (*n* = 25,435). A total of 407 respondents were excluded due to missing information concerning gender, educational achievement, generic frequency of drinking, or frequency of RSOD. The final sample included 12,512 women and 12,516 men from 18 locations. A question regarding the required quantity of alcohol needed to perceive impairments was asked in only 13 out of the 18 locations. In these locations, 4,604 respondents specified a quantity of alcohol needed to perceive impairments (see Table 1), while the majority of respondents did not, as most of them claimed not to experience any of the intoxication symptoms described in the question and were therefore not asked about the quantity of alcohol needed. Sample characteristics as well as the gender-specific threshold of alcohol intake to perceive impairments by location are presented in Table 1.

**Table 1:** Sample size, demographic characteristics and alcohol needed to perceive impairment by gender and location (*n* = 25,028).

Location	Sample size	Percent women	Mean age (SD)	Required quantity of alcohol needed to perceive impairments (grams of pure ethanol)^a^			
				Women (*n* = 1,745)		Men (*n* = 2,859)	
				Mean (SD)	*n*	Mean (SD)	*n*
**Austria**	2,969	49.0	41.2 (13.5)	n. a.		n. a.	
**Bulgaria**	2,628	49.0	41.6 (13.2)	n. a.		n. a.	
**Croatia**	1,166	45.0	40.6 (13.8)	105.0 (53.6)	78	186.5 (102.2)	231
**Denmark**	1,416	53.0	43.8 (13.7)	n. a.		n. a.	
**Estonia**	1,878	51.2	40.7 (13.0)	n. a.		n. a.	
**Finland**	1,343	48.8	41.7 (14.6)	81.4 (41.9)	229	120.1 (52.8)	401
**France**	1,397	48.9	42.4 (14.5)	49.7 (30.0)	71	65.4 (39.1)	126
**Greece**	1,365	48.6	41.1 (13.1)	46.8 (35.3)	92	65.5 (40.6)	194
**Hungary**	1,516	45.3	41.3 (14.5)	40.1 (24.7)	75	68.3 (48.2)	198
**Iceland**	673	51.6	40.1 (15.4)	n. a.		n. a.	
**Italy**	988	44.1	42.2 (13.3)	43.9 (24.4)	29	90.1 (59.9)	54
**Lithuania**	1,320	50.2	41.0 (13.5)	62.9 (14.9)	381	99.1 (32.3)	518
**Poland**	1,323	47.0	40.8 (13.2)	58.6 (29.6)	94	106.8 (69.9)	230
**Portugal**	1,038	43.2	40.4 (13.0)	51.3 (30.0)	25	98.0 (63.7)	68
**Spain**	1,326	46.4	40.8 (12.4)	56.6 (34.5)	193	76.0 (49.5)	306
**Spain-Catalonia**^b^	510	46.3	40.6 (11.9)	46.4 (23.9)	47	60.4 (22.8)	89
**Sweden**	1,322	50.4	40.9 (13.9)	63.1 (26.8)	243	87.2 (40.4)	299
**UK**^c^	850	49.8	42.0 (18.6)	46.4 (36.3)	196	56.0 (34.4)	153

Note.

^a^Alcohol needed to perceive impairments was not assessed in Austria, Bulgaria, Denmark, Estonia and Iceland;

^b^Spain-Catalonia = Spanish Autonomous Community of Catalonia;

^c^UK = United Kingdom of Great Britain and Northern Ireland;

n. a. = not applied.

### Statistical analysis

The prevalence of SI and RSOD were defined by the survey-weighted proportion of respondents who reported at least one episode of SI or RSOD in the past 12 months by location. Population-weighted averages of both the SI and RSOD prevalence estimates were calculated with population statistics obtained from the United Nations [16]. To compare the prevalence of both measures, a Spearman rank correlation at the aggregated level was calculated for the total sample (*n* = 25,028), and by gender (women: *n* = 12,512; men: *n* = 12,516).

For the subsample of individuals reporting any quantity of alcohol needed to perceive impairments, we ran regression analyses to investigate differences between the SI and RSOD measures. Survey weights were applied in all regression models, account for country-specific sampling bias. In a first regression model, the association of the difference between the frequency of SI events and RSOD (outcome) with the subjective threshold of alcohol needed to perceive impairments (predictor) was investigated using multinomial logistic regression analysis. The outcome variable distinguished between respondents reporting subjective intoxication less (‘1’; *n* = 1,299), similar (‘2’; *n* = 2,254) and more (‘3’; *n* = 1,051) frequently than they reported RSOD, based on the RSOD frequency measure, in the past 12 months. This means, for example, that a person who did not indicate subjective intoxication within the past year but indicated at least one RSOD would have been assigned to group 1. The quantity of alcohol intake needed to perceive impairments in hectograms of pure ethanol was used as predictor variable. Hectograms were used as the unit of measure to get more interpretable regression coefficients. The regression model was adjusted for gender, age, and the respondent’s location to account for cross-country variations in drinking behaviour. The variance explanation was estimated using McFadden R^2^ (Eq 1) [17], the quotient of the logarithms of the log likelihood function of the full model including the predictor variable (*L*_1_) and the log likelihood function under the empty model (*L*_0_):

$$R_{McFadden}^{2}\;=\;1-\frac{\text{ln}\;L_{1}}{\text{ln}\;L_{0}}$$ (1)

In a second regression model, we conducted a linear regression analysis to evaluate the explained variance of the difference between the required alcohol needed to perceive impairments and the gender-specific RSOD threshold value (outcome) by respondent’s characteristics (predictor variables; *n* = 4,544 [60 missing values for body weight]). The outcome variable was the difference between the individual threshold of the required quantity of alcohol needed in hectograms of pure ethanol minus the gender-specific RSOD threshold, which defines intoxication as drinking more than 40 grams of pure ethanol in women and more than 60 grams pure ethanol in men. Predictor variables were gender (women, men), age group (≤ 34 years, 35–49 years, ≥ 50 years), body weight in kg, and educational achievement (primary and lower secondary education, secondary education, higher education). Furthermore, indicators for alcohol consumption were included: past-year frequency of drinking, quantity of alcohol intake in grams of pure ethanol per drink day within the past 12 months, experiencing at least one episode of RSOD in the past 12 months, high-risk drinking defined by drinking more than 40 grams of pure ethanol for women or 60 grams of pure ethanol for men on a usual drink day [18], as well as the preferred type of alcoholic beverage (beer, wine, spirits). An individual’s preferred alcoholic beverage was determined by the beverage with the highest proportion of the beverage-specific alcohol intake out of the total alcohol consumption on a usual drink day. While RSOD represents drinking above a defined threshold in at least one episode in the past year, high-risk drinking refers to the consumption of alcohol above a threshold on each drinking day. Additionally, the respondent’s location was included as control variable. The variance explanation indicator R^2^ was estimated based on the residual sum of squares (RSS) and the total sum of squares (TSS; see Eq 2) [19]:

$$R^{2}\;=\;1-\frac{RSS}{TSS}$$ (2)

Variance explanation was determined separately for each predictor variable as well as cumulative over all predictors. All analyses were run using Stata 15.1 [20].

## Results

**Fig 1:**
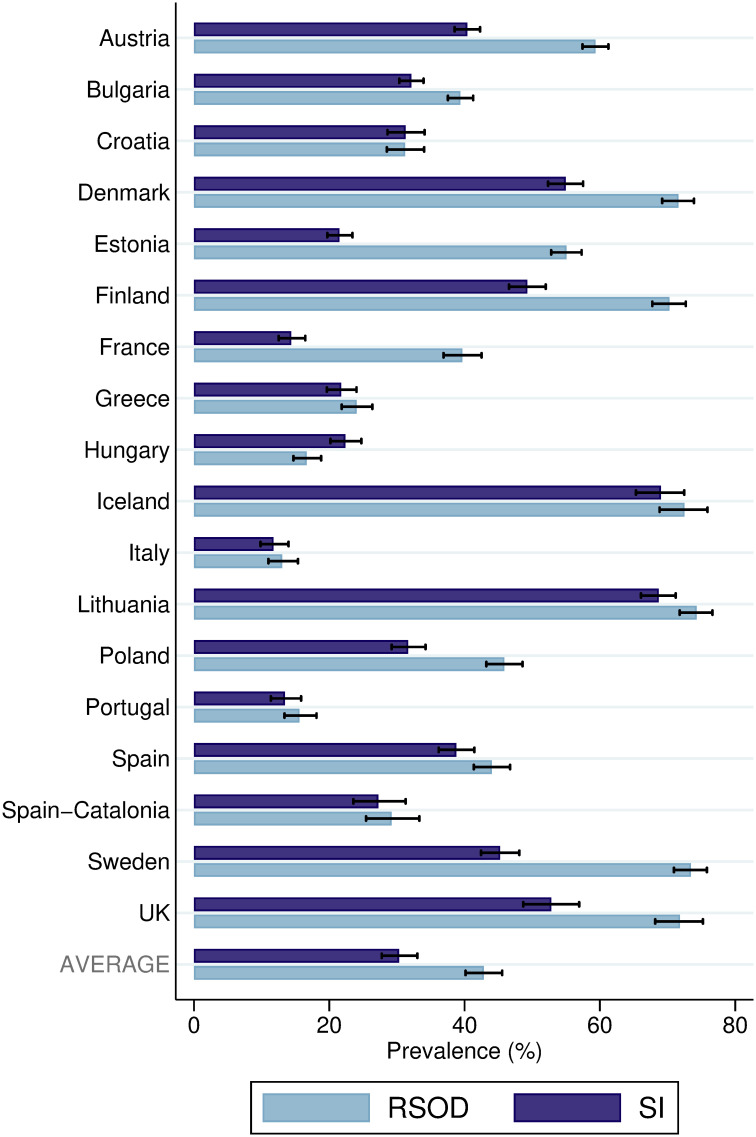
Prevalence and population-weighted average prevalence of RSOD and SI by location. Prevalence and population-weighted average prevalence are provided with 95% confidence intervals. At least one episode of RSOD (risky single-occasion drinking) or SI (subjective intoxication) within the past 12 months. Spain-Catalonia = Spanish Autonomous Community of Catalonia; UK = United Kingdom of Great Britain and Northern Ireland.

The prevalence of reporting at least one episode of SI or RSOD in the past 12 months are presented in Fig 1. On average, the prevalence based on SI was 12.5% lower than those based on RSOD. The greatest difference between RSOD and SI-based prevalence was observed in Estonia at 33.6%, whereas the smallest was in Croatia at 0.1%. Independently of measurement, the prevalence ranged from about 12% in Italy to almost 70% in Iceland and Lithuania. RSOD and SI-based prevalence were highly correlated (*r*_S_ = .86, *p* < .001). Even when gender was taken into account, there was a significant association between both estimates (women: *r*_S_ = .68, *p* = .002; men: *r*_S_ = .72, *p* = .001).

Multinomial regression analysis revealed that among those who provided information on past-year SI, differences between the frequency of SI and the frequency of RSOD were associated with the subjective threshold of alcohol intake needed to perceive impairments. Compared to respondents reporting equal frequencies of SI and RSOD, reporting fewer SI events than RSOD events was associated with a higher subjective threshold of alcohol needed to perceive impairments (RRR = 2.25, *p* < .001, 95% CI: 1.92 − 2.65). In contrast, reporting a higher frequency of SI than the frequency of RSOD was associated with a lower subjective threshold (compared to equal frequencies of SI and RSOD; RRR = 0.09, *p* < .001, 95% CI: 0.06 − 0.14). The predictor explained 7.7% of the variance in the difference between the frequency of SI and the frequency of RSOD.

Results from linear regression model which investigated the difference between the subjective threshold of alcohol needed to perceive impairments from the gender-specific threshold value of RSOD are displayed in Table 2. Being 50 years and older, having a secondary or higher education, preferring wine or spirits over beer drinking, and reporting high-risk drinking was significantly associated with a lower difference between the subjective quantity estimate and the RSOD threshold value, while being female, a higher body weight, more frequent drinking, a higher quantity of alcohol intake on a usual drink day, and the occurrence of RSOD were associated with a greater difference. In total, all predictors explained about 18% of the variance, with the occurrence of at least one RSOD within the past 12 months providing the greatest variance explanation (R^2^ = 6.3%). Other predictors such as high-risk drinking, age group, and preferred beverage type did not explain substantial variance.

**Table 2:** Linear regression model and variance explanation of demographic and sociodemographic characteristics and drinking patterns for the difference between the subjective threshold of alcohol needed to perceive impairments and the gender-specific RSOD threshold value (40 grams of pure ethanol for women and 60 grams of pure ethanol for men; outcome), n = 4,544^a^.

	Coef.	*p*	95% CI^b^	R^2^ (%)	cum. R^2^ (%)^c^
**Occurrence of at least one RSOD**^d^ **(past 12 months)**	0.34	< .001	[0.31; 0.38]	6.3	6.3
**Quantity of alcohol intake in grams pure ethanol per drink day (past 12 months)**	0.01	< .001	[0.01; 0.02]	6.0	12.3
**Body weight in kg**	0.005	< .001	[0.00; 0.01]	2.5	14.8
**Gender (Reference: Male)**					
**Female**	0.04	0.006	[0.01; 0.08]	1.1	15.9
**Past-year frequency of drinking**	0.09	< .001	[0.07; 0.11]	1.0	16.9
**Educational achievement (Reference: Primary and lower secondary education)**					
**Secondary education**	-0.12	< .001	[-0.17; -0.08]		
**High education**	-0.16	< .001	[-0.21; -0.11]	0.6	17.5
**High-risk drinking**	-0.51	0.001	[-0.80; -0.21]	0.3	17.8
**Preferred beverage (Reference: Beer)**					
**Wine**	-0.06	0.001	[-0.09; -0.02]		
**Spirits**	-0.06	0.002	[-0.09; -0.02]	0.2	18.0
**Age (Reference: ≤ 34 years)**					
**35–39 years**	-0.02	0.204	[-0.05; 0.01]		
**≥ 50 years**	-0.09	< .001	[-0.12; -0.05]	0.2	18.2

Note.

^a^Body weight was missings in 60 respondents;

^b^CI = Confidence interval;

^c^cum. = cumulative;

^d^RSOD = Risky single occasion drinking.

High-risk drinking was defined by drinking at least 40 grams (women) or 60 grams (men) of pure ethanol on a usual drink day. Model was adjusted for the respondent’s location.

## Discussion

The current article compared a subjective measure of intoxication with the more objective quantity measure of RSOD in order to evaluate their relative contributions in recording harmful drinking occasions from survey. Our results indicated a high correlation between the SI and RSOD measures for annual prevalence of drunkenness. However, the difference between both of these prevalence estimates varied across locations, with SI based prevalence being on average lower than the RSOD prevalence. At the individual level, the difference in the reported frequency of SI and the frequency of RSOD was associated with the subjective threshold of alcohol intake needed to perceive impairments. A higher subjective threshold was associated with a lower frequency of SI events compared to the frequency of RSOD. Demographic and sociodemographic characteristics, body weight, and drinking patterns were not able to explain considerable variance in the difference between the quantity of alcohol needed to perceive intoxication and the gender-specific RSOD threshold value. Based on our findings, we conclude that the SI measure may not offer substantial additional information beyond RSOD. However, what we cannot rule out is that the two concepts may differ in their predictive validity for the onset of an alcohol use disorder. Higher quantities needed to feel intoxicated may constitute the more relevant indicator as this measure is conceptually closely related to tolerance, which is a key feature of alcohol use disorders in different classification systems [21, 22]. This link may be best examined in prospective studies.

Limitations of the study must be taken into consideration. First, there is inherent bias in the reporting of the SI due to interpretation of intoxication [8, 12, 23]. Translation bias in the surveys was mitigated by the use of alternate phrases for intoxication rather than the words ‘intoxication’ or ‘drunkenness’. However, there is a noticeable huge variability in the intoxication threshold between and within countries, with variations of up to 80 grams of pure ethanol between Croatian women and men. Furthermore, the resulting blood alcohol concentration—an objective measure for intoxication—after consuming more than 100 grams of pure ethanol would lead to an acute intoxication with clinical relevance for ‘normal’ drinkers, rather than perceive impairments by alcohol. The huge variability in the intoxication thresholds provided by subjective reports of intoxication and biological plausibility causes the validity of these data to be questionable. Moreover, the restricted availability due to the optional nature of the survey item and a high non-response rate must be taken into account in the assessment of SI. Second, survey data is generally limited due to the (a) restricted representativeness of the population [24], and (b) high non-response rate, which can be related to an under-representation of certain groups of people such as individuals with low income or high levels of drinking [25]. Third, there is an undercoverage of reported alcohol consumption from self-report measures when compared to ‘real consumption’ estimates based on more reliable aggregate consumption data (e.g., from routine statistics such as taxation records or production) [26]. This undercoverage probably affected the evaluation of SI as well. Finally, only associations were investigated in our analyses and therefore interpretations of the directions of effects are not permissible.

Our results indicate a limited use for the SI measure in public health research. First, both the SI and RSOD measures were highly correlated, with the SI-based prevalence being 12.5% lower than the RSOD-based estimates. Second, the difference between both measures could not be well explained. Third, level of drinking and frequency of drinking occasions above a threshold has been well established as a determinant of morbidity and mortality independent of SI [27, 28]. To date, no risk relationships have been established for SI or the thresholds experienced. Finally, there is no easy way to correct for undercoverage in the reporting of alcohol use in the SI measure [e.g., 29, 30].

In conclusion, our results indicate that in epidemiological cross-cultural studies subjective measures of intoxication are not preferable to conventional RSOD measures, for the previously described reasons—e.g., bias due to differing perceptions of intoxication as a variable for determining alcohol-attributable burden, or as a measure with public health importance. Of course, there may be other uses for the SI measure, such as the possibility of a better understanding human social cognition. For issues concerning public health, however, RSOD seems to be the best measure available to evaluate high volume drinking.
